# “GO” to move toward dementia‐friendly communities: A pilot study

**DOI:** 10.1002/brb3.3581

**Published:** 2024-06-07

**Authors:** Ai Iizuka, Chiaki Ura, Mari Yamashita, Koki Ito, Miyuko Yamashiro, Tsuyoshi Okamura

**Affiliations:** ^1^ Research Team for Social Participation and Healthy Aging, Tokyo Metropolitan Institute for Geriatrics and Gerontology Itabashi‐ku Tokyo Japan; ^2^ Research Team for Promoting Independence of the Elderly, Tokyo Metropolitan Institute for Geriatrics and Gerontology Itabashi‐ku Tokyo Japan

**Keywords:** aging, cognitive activity, community care, dementia, mild cognitive impairment

## Abstract

**Background:**

To the creation of mutual aid relationships among people with cognitive decline is important in aging societies. This study aimed to develop and examine the feasibility of a GO Program in which older adults, with experience in playing GO, support the learning of GO by older adults with cognitive decline and other barriers to social participation, which in turn reduces social isolation and creates opportunities for older adults to use their role.

**Methods:**

This single‐arm intervention study was conducted in Tokyo, Japan. Introductory GO classes were held for 10 participants who had never played GO (beginners) and 10 participants who had (supporters) once a week for an hour, for a total of 12 sessions. Supporters and beginners were paired to solve problems and play games. We assessed the feasibility of the program and its effects on mental health social network, and cognitive function.

**Results:**

Cognitive test scores were at the mild cognitive Impairment level for beginners as well as for supporters. Satisfaction with the program was high, with an overall class attendance rate of 99.1% and none leaving the program. No significant changes were observed over time for beginners in each measurement; however, there was a significant improvement in the Mini Mental State Examination‐Japanese scores for supporters (*p* < .05).

**Conclusions:**

The results suggest that this program could contribute to the creation of mutual aid relationships among older adults with cognitive decline; even if they have mildly declined cognitive function, they can still play an active role in society. Moreover, creating such opportunities may positively impact cognitive function.

## Graphical abstract

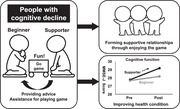

## 1 INTRODUCTION

The increasing number of people with dementia is a major problem in aging societies, and preventing or delaying the onset of dementia is a public health priority (Livingston et al., 2017, 2020). In Japan, the prevalence of dementia is expected to reach 7 million by 2025 (Health & Welfare, Bureau for the Elderly, Ministry of Health, Labor & Welfare, 2018), and the “Outline for Promotion of Dementia Policy,” the government's policy for dementia countermeasures in 2019, emphasizes the importance of “living‐well with dementia and prevention of dementia” (Ministry of Health, Labour & Welfare, 2021).

The risk of developing dementia is reported to be decreased by maintaining appropriate lifestyle habits (Livingston et al., 2017, 2020). Improving cognitive reserve through exercise, education, intellectual activity, and social interaction is one strategy to prevent dementia (Stern, 2002, 2009). Specifically, the COVID‐19 pandemic has negatively impacted the health of older adults by limiting their interaction with people, leading to cognitive decline (Corbett et al., 2023; Li et al., 2023). Social interaction provides access to new ideas, information, and diverse visual and auditory stimuli (Mische & White, 1998). Moreover, the density and size of these social interactions are associated with the volume of specific brain regions related to dementia (Chalfont et al., 2020; Kwak et al., 2018). Many previous studies have shown that the risk of developing dementia reduces with greater social interaction (Crooks et al., 2008; Evans et al., 2019; Kelly et al., 2017; Penninkilampi et al., 2018). However, although it is possible to reduce the risk, it is difficult to completely prevent dementia. Furthermore, the development of a fundamental cure for dementia continues to be challenging. Therefore, it is necessary to create a system that supports people with dementia in the community.

Based on this, a community development effort known as a “Dementia Friendly Community (DFC)” is spreading (Hung et al., 2021; Wu et al., 2019). DFCs are communities where people with dementia feel understood, respected, and supported by those around them and feel more confident in participating in and contributing to social activities. In Japan, policies aimed at promoting successful DFCs, as well as activities organized by companies, nonprofit organizations, and community organizations, have been gradually expanding and spreading awareness since the 2010s (Tsuda et al., 2022). Our research team has been conducting epidemiological and action research since 2016, establishing a community center within the Takashimadaira housing complex in Itabashi ward, Tokyo, to build an urban model of DFCs (Okamura et al., 2020; Ura et al., 2021). The study found that many people in the community with cognitive decline or dementia have not been diagnosed at a hospital (Ura, Okamura, Inagaki, et al., 2020), have lost touch with society, and live in isolation. One‐third of these people leave the community over a 3‐year period because of death, hospitalization, or residential care.

This community center offers monthly activities with the strategic board game “GO” for older adults. GO is a historical and cultural board game that originated in China around 2000 B.C. and was introduced in Japan around 700 A.D. In recent years, the GO population has increased in Western countries (Cho, 2015; The Nihon Ki‐in, 2023). The GO activities at this community center have been well received by the residents, and group activities run by the participants themselves are held on a regular basis.

However, there is almost no participation of older adults with dementia and cognitive decline, who are easily isolated from the community. A telephone survey conducted in the region during the COVID‐19 pandemic in 2020 showed that older adults with cognitive decline had significantly fewer opportunities to go out, exercise, and interact with others by phone and email during the pandemic compared to older adults who used community centers (Ura, Okamura, Sugiyama, et al., 2020). Therefore, we considered that creating a place where older adults with cognitive decline can meet other older adults who are already participating in GO classes at the community center and form a supportive relationship through the game would be helpful for living well with dementia. Thus, older adults with cognitive decline will have opportunities for social participation, and people with GO experience will play an active role in supporting others. However, is this really possible? The research question for this study was as follows: Can GO be used as a tool for older adults in the community and people with cognitive decline to meet, interact, and create supportive relationships?

First, to determine the suitability of GO as an activity that creates supportive relationships among older adults and allows them to play an active role, interviews were conducted with individuals who started playing GO at an older age. Interview questions aimed at finding out why they started playing GO and what it means to them (Iizuka et al., 2023). The results showed that GO is not only a game but also a learning tool that promotes inquisitiveness for older adults, a psychological stabilizing tool that relieves loneliness, and a social tool for inviting and teaching each other. In addition, as the game involves intellectual stimulation that includes complex strategies and requires continuous learning, the prevention of cognitive decline is a motivating factor for participation. Our previous study also reported that GO has an inhibitory effect on cognitive decline, which is more pronounced when played with others face‐to‐face than when played alone (Iizuka et al., 2019), learning GO skills increases brain activity in the frontal lobe, basal ganglia, and so on (Iizuka et al., 2020), and that even older adults with dementia who have never played the game before can learn and enjoy it because of its simple rules (Iizuka et al., 2018). Given this, GO is a tool that is favorably accepted by both cognitively impaired and healthy older adults. By providing these opportunities, we hypothesized that GO could be used as a tool for both to meet and interact with each other in a new way and create supportive relationships. However, there is no research on how to use GO in the real world to create such relationships.

This study aimed to develop and examine the feasibility of a GO program in which older adults with experience in playing GO support the learning of GO by older adults with cognitive decline and other barriers to social participation and to explore its effects on cognitive function, mental health, and social networks. Specifically, the effects of the GO program on the supporters and the supported (beginners) were examined in a single‐arm intervention study.

## 2 METHODS

### 2.1 Participants

This study was conducted from July 2021 to May 2022 in the Itabashi Ward, Tokyo. The participants were older adults with experience in learning GO (supporters) and older people with cognitive decline or social isolation who had no experience in GO and wished to participate in the GO class (beginners). The inclusion criteria were as follows: (1) age 65 years or older and (2) independence in activities of daily living. Supporters are those who have (3) experience in learning GO, whereas beginners are those who have (4) no experience in learning GO, and (5) a Japanese version of the Montreal Cognitive Assessment Scale (MoCA‐J) score of 26 or less (Fujiwara et al., 2010) or the Lubben Social Network Scale‐6 (LSNS‐6) score of 12 or less (Gray et al., 2016; Kurimoto et al., 2011) were added. The purpose of this study was to create a place where people who have difficulty getting involved in society due to cognitive decline, or people who are already socially isolated, can make new social connections and support each other through GO. Therefore, we included a cognitive test and an index of isolation in the inclusion criteria. The exclusion criteria for supporters and beginners were (1) those undergoing treatment for acute illness, (2) those unable to participate in the intervention on their own, and (3) those with severe visual or hearing impairment.

The supporters were recruited from GO classes held at the community center and a voluntary GO group in the area. Beginners were recruited by distributing flyers to users of the regional center and referrals from staff at nearby community support centers and dementia cafés. A research briefing session was held to explain the purpose, methods, ethical considerations, and other details of the study. Finally, 20 people—10 supporters and 10 beginners—who met the inclusion criteria were included in this study.

### 2.2 Intervention

The intervention consisted of 12 weekly 60‐min introductory GO classes (GO programs) held at the community center from January 2022 to April 2022. Based on a previous program we developed and examined the effectiveness of using GO with healthy older adults, we modified the program in this study to make it easier for older people with cognitive decline to understand and learn game skills in few steps. In addition, because many of the supporters had never taught GO to beginners before, a training session was held once before the intervention to teach tips, caution, and manners of giving advice. Each session consisted of a lecture, an exercise, and a game. Supporters and beginners participated in pairs, and supporters were encouraged to cooperate with beginners by providing advice and assistance for solving problems and playing games. In addition, the beginners worked on homework after each class, which consisted of 2–4 GO exercises per day for 10 min. In addition, supporters gave the beginner advice on how to solve the homework over the phone, once per week.

### 2.3 Measures

Program assessments were conducted twice at the community center: before (baseline) and after the program (3 months later). Cognitive tests and individual interviews were conducted by a clinical psychologist or trained staff member.

#### 2.3.1 Baseline characteristics

Baseline characteristics, such as age, sex, educational background, employment status, presence or absence of family members living together, and independent activities of daily living (Tokyo Metropolitan Institute of Gerontology Index) (Koyano et al., 1991), were investigated. The participants were also screened using the MoCA‐J, a screening test for mild cognitive impairment (MCI), and the LSNS‐6, a measure of social networking.

#### 2.3.2 Feasibility

As for the feasibility of the program, attendance rates from each session and dropout rates from the program were recorded, and a self‐administered questionnaire was used to assess program satisfaction, willingness to continue playing GO, and to continue supporting beginners.

Program satisfaction was assessed in terms of scores (on a scale of 0–100), and the reasons for the scores were interviewed individually (in particular, the reasons for the reduction in points). Willingness to continue playing GO (beginners only) and willingness to continue beginner support (supporters only) were rated on a 4‐point scale: “agree, somewhat agree, somewhat disagree, and disagree.” In addition, the participants were individually interviewed regarding their impressions of the GO classes.

#### 2.3.3 Cognitive function

The Mini Mental State Examination‐Japanese (MMSE‐J) (Folstein et al., 1975; Sugishita et al., 2016) and the MoCA‐J were used to assess cognitive function. MMSE‐J is a screening tool for dementia. The maximum score on the MMSE is 30, and the cutoff score for screening for dementia is 23/24. The MoCA‐J is a brief cognitive screening tool for MCI, with a maximum score of 30 and a clinical cutoff score of 25/26.

#### 2.3.4 Mental health

Mental health was assessed using the Japanese versions of the WHO‐5 Mental Health Status Table (WHO‐5‐J) (Awata et al., 2007) and the Japanese version of the three‐item UCLA Loneliness Scale (Arimoto & Tadaka, 2019). The WHO‐5‐J is a five‐item scale that assesses mood states in daily lives. The scores range from 0 to 25, with higher scores indicating better mental health. The three‐item version of the UCLA Loneliness Scale is a psychological scale that measures loneliness, with scores ranging from 0 to 12, and higher scores indicating greater feelings of loneliness. In this study, Cronbach's alpha values for the WHO‐5‐J and UCLA Loneliness Scale were 0.623 and 0.773, respectively.

To further interpret the results of these questionnaires, the participants were interviewed individually about their mood and life changes before and after participation in the program.

#### 2.3.5 Social network

The LSNS‐6 is a screening scale for social isolation that is used as a measure of social networks. The scores range from 0 to 30, with higher scores indicating greater social networks and scores below 12 indicating social isolation. In this study, Cronbach's alpha for the LSNS‐6 was 0.929.

To further investigate the changes in social networks as a result of program participation, we asked whether any new acquaintances in the program interacted outside the program.

### 2.4 Statistical analysis

Wilcoxon‐signed‐rank tests were conducted to determine the intervention effects of the program on the pre‐ and posttest scores for each assessment item. In addition, stratified analyses were conducted for supporters and beginners to examine the differences in the intervention effects between them.

### 2.5 Ethical approval

Ethical approval was obtained from the Institutional Review Board and Ethics Committee of the Tokyo Metropolitan Institute of Gerontology (Approval No. R21‐067). We explained the purpose, methods, and ethical considerations of this study and obtained written informed consent from each participant based on the Declaration of Helsinki before enrolment.

## 3 RESULTS

### 3.1 Participant characteristics

The participants’ basic characteristics are shown in Table 1. One beginner refused to complete the posttest; therefore, a total of 19 participants (10 supporters and 9 beginners) who ultimately completed the program were included in the assessments. The mean age was 80.3 ± 5.0 years (mean ± standard deviation), 68% were female, and more than 90% were not employed. At the time of creating the study design, supporters were assumed to be those with no cognitive decline, but upon actual assessments, both supporters and beginners were found to be at the MCI level (The mean MoCA‐J score of the supporters was 23.3 ± 2.9 and the mean score of the beginners was 22.2 ± 3.3). The mean LSNS‐6 score of the beginners was 10.7 ± 6.4 points, and they were considered socially isolated.

**TABLE 1:** Baseline characteristics of all participants.

		All participants (*n* = 19)	Supporters (*n* = 10)	Beginners (*n* = 9)
Age, mean (*SD*)	Years	80.3 (5.0)	80.6 (4.7)	80.1 (5.2)
Gender (female)	％	68.4	80.0	55.5
Years of education (≦12 years)	％	52.6	50.0	55.5
Current occupational status (unemployed)	％	94.7	90.0	100.0
Family living together (solitary life)	％	42.1	30.0	55.5
MoCA‐J, mean (*SD*)	score (0–30)	22.7 (3.2)	23.3 (2.9)	22.2 (3.3)
LSNS‐6, mean (*SD*)	score (0–30)	12.4 (6.1)	13.8 (5.5)	10.7 (6.4)

*Note*: Results for Mann–Whitney *U* test.

Abbreviations: LSNS‐6, Japanese version of the abbreviated Lubben Social Network Scale.; MoCA‐J, Japanese version of the Montreal Cognitive Assessment.

### 3.2 Feasibility

Program attendance was very high (99.1%), and there were no dropouts from the intervention. Table 2 shows the program satisfaction scores and reasons given by each participant. The average score was 90 out of 100, and the reasons for the scores were opinions about the contents of the GO program, such as “the instructor's explanations were easy to understand” and “the program content was good.” The most common reason for the reduction scores was that “the time for lectures and games was too short,” and 2 out of 19 participants also commented about the interactions, saying that “I wanted to interact with younger people” and that “I could not deepen interactions with others.”

**TABLE 2:** Program satisfaction scores and reasons.

	Participants	Scores	Reason for scoring
Supporters	A	100	I enjoyed taking the program. The content was easy to understand
	B	90	The reason why I deducted the points was he didn't respond to my conversation
	C	80	The number of courses was just fine, but I wanted more time for games and commentary
	D	90	I wanted a little more time for the game
	E	80	I didn't have enough time to practice
	F	100	I liked the way they did it
	G	100	The explanation was detailed and easy to understand
	H	85	I didn't know how to determine our Go ability
	I	80	I need to get better at Go for teaching others
	J	98	I didn't have enough time to play the game. I felt pressed for time
Beginners	a	100	I was worried at first, but I understand now
	b	80	I wanted to be taught the same thing over and over again
	c	80	The game time was too short. I wanted to look back on the process of my strategy
	d	95	I wanted to play more Go
	e	70	Many of us had never met each other before, so i didn't get to interact with them
	f	100	The program was easy to understand for beginners
	g	90	I wanted more time for the program
	h	80	I wished there was more interaction with the younger generation as well as the same generation
	i	100	I wanted more time for the program

**FIGURE 1:**
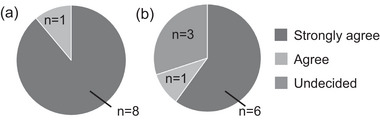
Willingness of beginners to continue playing GO and willingness of supporters to continue supporting these beginners: (a) willingness of beginners to continue playing GO and (b) willingness of supporters to continue supporting the beginners.

The results of the questionnaire regarding the beginners’ willingness to continue playing GO and the supporters’ willingness to continue supporting the beginners are shown in Figure 1. Regarding the willingness of beginners to continue playing GO, eight respondents answered “strongly agree” and one “agree,” indicating that all participants were willing to continue playing the game. Regarding the willingness of supporters to continue supporting beginners, one respondent answered “strongly agree,” six answered “agree,” and three answered “undecided,” indicating that 70% of respondents were willing to continue the program.

The results of the interviews regarding their impressions of the program are presented in Table 3. Overall, beginners made positive comments about learning a new game of GO, such as how interesting it was and how easy it was to understand the instructor's explanations. Supporters expressed their impressions from the standpoint of “teaching,” such as their own lack of ability and the tension they felt when they were on the teaching side, whereas at the same time, they felt that it was beneficial to review their knowledge of GO. On the other hand, both the beginners and supporters mentioned the short time and duration of the program as negative comments.

**TABLE 3:** Feedback on the program.

	Participants	Feedback on the program
Supporters	A	I enjoyed the program and the explanations were easy to understand. I was nervous to be a supporter
	B	I was not sure if I could be a supporter, but I enjoyed it
	C	Everyone seemed to be having fun and the atmosphere was friendly
	D	The explanations were easier to understand than before, and I could re‐learn some things
	E	The instructor's explanations were easy to understand. Supporters had difficulty because the foundations were not solid
	F	It was good for beginners. It was also good for beginners to be able to review important points with their supporters
	G	I learned something new every time I took the program. I wanted to extend the time and take my time
	H	The textbook was easy to understand. I felt it was hard to consider the opponent in a match, considering their abilities
	I	I felt I was not strong enough. I was nervous about being a supporter
	J	It was a fun program
Beginners	a	I enjoyed it more than I expected
	b	I enjoyed it, but I felt it was more difficult each time I tried the program
	c	I became more interested in Go and enjoyed participating. I want to practice and become more skilled
	d	I was anxious and nervous at first, but once I understood how to do it, I was happy and had a lot of fun
	e	It was my first time playing Go, but I enjoyed the devoted guidance and advice of the people around me
	f	I learned the basics of Go and enjoyed working on it. I thought it was promising for dementia prevention
	g	The explanations were easy to understand and I enjoyed taking the course. I was nervous and tired of playing games
	h	Explanations were detailed and easy to understand
	i	It was the first time in my life I played Go, and it was wonderful

### 3.3 Intervention effects

Pre‐ and post‐intervention scores for cognitive function, mental health, and social networks, as well as the results of the Wilcoxon signed‐rank test, are shown in Table 4.

**TABLE 4:** Results before and after intervention for the main evaluation items.

		All participants (*n* = 19)			Supporters (*n* = 10)			Beginners (*n* = 9)		
		Before	After	*p* ^a^	Before	After	*p* ^a^	Before	After	*p* ^a^
WHO‐5‐J	Score (0–25)	15.7 (4.2)	15.6 (4.5)	.96	16.0 (3.7)	16.6 (3.7)	1.00	15.3 (4.6)	14.5 (5.1)	1.00
UCLA	Score (3–9)	4.1 (1.6)	4.0 (1.7)	.54	4.0 (1.7)	3.2 (0.6)	.10	4.2 (1.3)	4.8 (2.1)	.25
LSNS‐6	Score (0–30)	12.4 (6.1)	11.2 (6.1)	.46	13.8 (5.5)	13.6 (5.0)	.81	10.7 (6.4)	8.6 (6.1)	.48
MMSE‐J	Score (0–30)	27.7 (1.8)	28.2 (1.9)	.16	27.8 (1.4)	29.2 (0.7)	.01^*^	27.6 (2.2)	27.2 (2.3)	.42
MoCA‐J	Score (0–30)	22.7 (3.2)	23.5 (3.3)	.19	23.3 (2.9)	24.5 (2.4)	.15	22.3 (3.3)	22.5 (3.8)	.77

Abbreviations: LSNS‐6, Japanese version of the abbreviated Lubben Social Network Scale; MMSE‐J, Japanese version of the Mini‐Mental State Examination; MoCA‐J, Japanese version of the Montreal Cognitive Assessment; USLA, Japanese version of the three‐item UCLA Loneliness Scale; WHO‐5‐J, Japanese version of the World Health Organization‐Five Well‐Being Index.

^a^ Results for Wilcoxon signed‐rank sum test.

* *p* < .05.

#### 3.3.1 Cognitive function

There were no significant changes in the participants’ cognitive test scores before and after the intervention (all *p *> .05) (Table 4). However, analyses stratified by supporters and beginners revealed that the MMSE‐J scores significantly improved post‐intervention (*p* < .05). In contrast, beginners showed no significant changes in MMSE‐J scores.

#### 3.3.2 Mental health

There were no significant changes in mental health over time for either the participants as a whole or in the stratified analysis by supporters and beginners. In the individual interviews, there were positive comments, such as feeling better and more positive, having more fun, having a better rhythm in life, and broadening one's horizons, but there was a comment from a supporter, such as “I felt depressed when my pair partner did not respond” (Table 5).

**TABLE 5:** Changes in mood and life before and after the program.

	Participants	Changes in mood and life before and after the program
Supporters	A	I have more energy in my life. I looked forward to taking the course
	B	I want to win, and I want to be strong
	C	By teaching others, I learned how narrow‐minded I am
	D	I was going to quit Go, but I wanted to continue a little longer. I have more time to play Go
	E	I can now use my time more effectively. I got to talk to people I'd never met before
	F	The same as before
	G	I am more efficient at cleaning up and have more time to study Go
	H	I can now hear what the other person is saying on the phone
	I	The same as before
	J	The same as before
Beginners	a	I have something to look forward to once a week, and it makes me feel better
	b	I look forward to interacting with people through taking courses, calling supporters, and playing Go
	c	My perspective was broadened by borrowing books and watching TV about Go
	d	I have more hobbies and I'm busier. I started to look back on games and practice
	e	I'm getting more positive
	f	I'm becoming more positive and cheerful. I have a lot more energy in my daily life
	g	I started playing games with my husband instead of watching TV at night
	h	The same as before
	i	I have more time for fun, but the same as before in my mood or life

#### 3.3.3 Social network

There were no significant changes in the social networks over time for either the participants as a whole or in the stratified analysis by supporters and beginners. In addition, only 26.3% of the participants interacted with new acquaintances outside of the course.

## 4 DISCUSSION

This study aimed to develop and examine the feasibility of a GO program and its effects on cognitive function, mental health, and social network exploration. The results of this study indicated that the program was feasible and could contribute to the social participation of older adults with cognitive decline and the creation of opportunities for them to play an active role in the community. In addition, the program has the potential to improve supporters’ cognitive functions. Therefore, GO could be considered a valuable tool for older adults in the community, and for people with cognitive decline, to meet, interact, and create supportive relationships.

Regarding the feasibility of the program, the attendance rate, program satisfaction, willingness to continue with GO, and beginner support were high, and none dropped out, suggesting that the program is feasible for development in the community. In particular, there were many positive comments about the GO teaching methods and program content, which may have influenced the participants’ satisfaction and willingness to continue GO or support beginners. However, both the beginners and supporters mentioned the short time and duration as negative comments. Because of this, only 26.4% of the participants developed new contacts outside of the program while expressing a desire for more opportunities to inter act with each other. Based on the above discussion, we consider that extending the duration of the program, taking time for self‐introductions, and incorporating team competitions to increase the elements of exchange are areas for improvement in the future development of the program. A comment regarding being troubled by the reaction of the partner was also observed, which indicated the need to consider the compatibility of the participants when pairing them and devising combinations.

Regarding the effects of the intervention, the results indicated that the program may improve the global cognition of supporters. Given that learning new skills has previously been shown to improve cognitive function (Park et al., 2014), and that the results of previous studies show that learning GO improved the cognitive function of novice players (Iizuka et al., 2018, 2019, 2020), we expected the program to be effective in improving the cognitive function of beginners. However, the results showed that the program may have been effective in improving the cognitive function of the supporters. This may indicate that the act of “teaching someone a skill that one has acquired oneself” has a strong impact on cognitive function. In a previous study, it was shown that some cognitive functions were improved by providing learning support to elementary school children. The results of this study support these findings (Carlson et al., 2008). During the program, each supporter was often seen working through trial and error on how to provide advice, help, and hold back when playing games, which may have had a positive effect on their cognitive function.

On the other hand, there was no effect of the program on the cognitive function of beginners. There are two possible reasons for this. First, as shown in our previous study, the GO intervention had an effect on visual working memory, but not on global cognition. It is considered that even if the interactional component of the GO classes was enhanced by the supporter's assistance, as in the present study, the intellectual stimulation was not large enough to have an effect on global cognition. In addition, the short‐term supporter‐assisted advice might not help beginners acquire advanced GO skills and had little impact on their global cognition. If a long‐term intervention is used and beginner understanding is promoted, there is a possibility that a certain effect can be observed.

Thus, it is possible that the cognitive function of the teacher increased more than that of the person being taught. This is a behavior that is more difficult to teach and therefore more cognitively stimulating. Therefore, it has a greater potential for improving cognitive function. This suggests that helping older adults may not only be an action of high moral value (i.e., doing something good to help others) but may also provide the practical value of improved cognitive function. Although it is difficult to generalize these results, they do suggest a positive aspect to helping each other.

Interestingly, the cognitive function of the participants (beginners and supporters) was at the MCI level. Usually, people with cognitive decline find it difficult to learn new things and receive assistance rather than support, and often lose their active role in society. However, the MCI‐level participants in this study became supporters and completed beginner support. This indicates that even those with mildly declined cognitive function can play an active role in society. Takehara described that in order to realize living well with dementia, it is necessary to dispel the labels, prejudices, and stigmas that come with a decline in cognitive function. That is, such individuals are finished and cannot understand anything (Takehara, 2022). The results obtained in this study may help to eliminate such prejudices. Furthermore, this study showed that creating such opportunities may have a positive impact on cognitive function. The finding that contributing to society may help maintain one's own health as well as influence the motivation of people with cognitive decline to participate in society themselves.

Although an intervention effect was observed on cognitive function, there were no significant changes in mental health or social activities. It is possible that cognitive function was affected because the participants played GO not only during the program but also outside the program, such as while doing their homework; however, the intervention may not have had a significant effect on mental health and social networks. Activities that are effective in maintaining and improving mental health regardless of cognitive decline or isolation include music, handicrafts, painting, and gardening (Abbing et al., 2018; Burt & Atkinson, 2012; Pöllänen & Weissmann‐Hanski, 2019; Van Der Steen et al., 2017). Unlike these activities, which appeal to the senses, games such as GO always produce a winner and a loser. The sense of burden related to losing can cause mental stress, which may impact the improvement of mental health. In addition, supporters took care to ensure that the paired beginners could learn comfortably, and in one case, there was a realization, such as in “I learned how narrow‐minded I am by teaching others,” which seems to have placed a considerable mental burden on the participants. However, in the interviews, there were many comments such as “I feel better” and “I feel more positive,” and it is possible that a long‐term intervention would result in changes in mental health and social networks.

As described before, we have now developed a program that enables active interactions between people with cognitive decline and older individuals within the community. The feasibility and effectiveness of this program have been demonstrated. In recent years, it has been reported that some characteristics of successful DFCs approaches are the creation of social networks that are actively available to people with cognitive decline, and the abundance of social support that is exchanged through neighborhood networks (Health & Global Policy Institute, 2019; Wiersma & Denton, 2016). In Japan, the frequency and duration of previously predominant community activities such as neighborhood associations and festivals have been reduced. It has been suggested that alternative volunteer groups and community salons may serve as a basis for DFCs (Tsuda et al., 2022). Another study suggests that interventions using a health promotion approach may have merit in creating DFCs (Kawachi & Berkman, 2014). However, few academic studies have examined the effectiveness of specific initiatives. This study may serve as a pioneering study in terms of program development, and in verifying the effectiveness that could contribute to the creation of DFCs. In addition, the finding that older adults helping each other is beneficial to their health will promote the creation of a compassionate community and lead to the creation of DFCs.

There are several limitations to this study. First, because this was a pilot study and the sample size was small, caution must be exercised in interpreting the results. To examine the intervention effects of the program in detail, the results of this study should be used to define the main outcome, and further research should be conducted. Second, there were many female participants and few male participants. As most GO enthusiasts are older men, it is perceived that GO is a tool that is relatively easily accepted by men; however, in future, it is necessary to consider ways to include men who are inexperienced in GO. Third, screening was conducted using the MoCA‐J and LSNS‐6 to select beginners; however, relatively active people participated. One of the objectives of this program was to create a place for people who have difficulty participating in society because of cognitive decline. The recruitment method used in this study may make it difficult to obtain the participation of older adults who tend to be isolated and shut‐in. In future, it will be necessary to consider ways to incorporate people with such tendencies. Finally, this is a short‐term study with no follow‐up. A follow‐up study is required to examine the changes in health status of the participants, and how they spent their time after the study ended. This would clarify whether the program truly contributed to building DFCs.

Although there are some limitations, there were also achievements such as the creation of relationships during the program, which although small in number, developed outside the program, and that GO became a new reason for living and helped relieve anxiety and worries in daily lives. Furthermore, individuals with cognitive decline can use the skills they have acquired to support others is a new finding in the field of dementia research. This program can be disseminated at community salons and other places, either in other areas or in the same area where the study was conducted. Additionally, it can create opportunities for older adults who participated in the study as beginners to contribute to society by shifting from being supported by others to helping others. Based on this pilot study, further research will be conducted, and the program will be implemented in various regions in the future.

## 5 CONCLUSIONS

The feasibility of the GO program was high, suggesting that it could contribute to the development of mutual‐support relationships among individuals with cognitive decline. The fact that people with cognitive decline supported beginners and succeeded indicates that even those with mild cognitive decline can play an active role in society and that providing such opportunities may have a positive impact on cognitive function. In future research, we plan to improve the program based on the findings of this study.

## AUTHOR CONTRIBUTIONS

**Ai Iizuka**: Conceptualization; investigation; funding acquisition; writing—original draft; formal analysis; methodology; project administration. **Chiaki Ura**: Conceptualization; investigation; methodology; writing—review and editing; project administration; supervision. **Mari Yamashita**: Investigation; methodology; validation; writing—review and editing; formal analysis; supervision. **Koki Ito**: Investigation; formal analysis; project administration; writing—review and editing; data curation. **Miyuko Yamashiro**: Investigation; writing—review and editing; data curation. **Tsuyoshi Okamura**: Conceptualization; methodology; supervision; resources; writing—review and editing.

## CONFLICT OF INTEREST STATEMENT

The authors have no potential conflicts of interest to disclose.

### PEER REVIEW

The peer review history for this article is available at https://publons.com/publon/10.1002/brb3.3581.

## Data Availability

The datasets used and analyzed during the current study are available from the corresponding author on reasonable request.
